# A novel redox signalling axis mediates nicotine-induced podocyte injury

**DOI:** 10.3389/fphar.2026.1916699

**Published:** 2026-08-13

**Authors:** Mohammad Atiqur Rahman, Rishith Yellu, Varnika Pavirala, Sayantap Datta, Saisudha Koka, Krishna M. Boini

**Affiliations:** Department of Pharmacological and Pharmaceutical Sciences, College of Pharmacy, University of Houston, Houston, TX, United States

**Keywords:** inflammasome, membrane raft, nicotine, podocytes, smoking

## Abstract

**Introduction:**

Nicotine has been shown to induce Nlrp3 (nucleotide-binding oligomerization domain-like receptor family pyrin domain-containing 3) inflammasome activation, thereby contributing to podocyte injury. However, the molecular mechanisms underlying nicotine-induced Nlrp3 inflammasome activation and subsequent podocyte dysfunction remain largely unknown. The present study investigated whether membrane raft (MR)-associated redox signalling plays a critical role in nicotine-induced Nlrp3 inflammasome activation and podocyte injury.

**Methods and Results:**

Nicotine treatment induced membrane raft clustering in podocytes in a dose-dependent manner. Upon nicotine stimulation, the NADPH oxidase subunits gp91^phox^ and p47^phox^ were recruited and aggregated within membrane raft clusters, leading to the formation of a membrane raft redox signalling platform. The formation of this signalling platform was significantly inhibited by pretreatment with the membrane raft disruptor methyl-β-cyclodextrin (MCD), the NADPH oxidase inhibitor diphenyleneiodonium (DPI), or the acid sphingomyelinase (Asm) inhibitor amitriptyline. Nicotine also markedly enhanced the colocalization of Nlrp3 with ASC and caspase-1, indicating inflammasome assembly, and significantly increased caspase-1 activity, IL-1β production, desmin expression, podocyte permeability, and apoptosis compared with control cells. These effects were significantly attenuated by pretreatment with MCD, DPI, the caspase-1 inhibitor WEHD, or amitriptyline. Furthermore, immunofluorescence analysis demonstrated that nicotine significantly reduced podocin expression, whereas pretreatment with MCD, DPI, WEHD, or amitriptyline largely preserved podocin expression.

**Conclusion:**

Our findings demonstrate that membrane raft-associated redox signalling serves as a critical upstream mechanism driving nicotine-induced NLRP3 inflammasome activation and podocyte injury.

## Introduction

Nicotine addiction remains a major global public health challenge, with detrimental effects that extend beyond the cardiovascular and nervous systems to significantly impair renal health (Mishra et al., 2015; Benowitz and Burbank, 2016; Rahman et al., 2026). Tobacco use, the primary source of nicotine exposure is a leading cause of preventable morbidity and mortality and has been increasingly recognized as an important risk factor for the development and progression of chronic kidney disease (CKD) (Jain and Jaimes, 2013; Wu et al., 2021). Growing evidence indicates that nicotine contributes to CKD through multiple, interconnected pathogenic mechanisms (Rezonzew et al., 2012). In particular, nicotine promotes oxidative stress and inflammation, two key drivers of renal injury that disrupt glomerular and tubular homeostasis (Caliri et al., 2021). These deleterious effects are further amplified by nicotine-induced vasoconstriction, which reduces renal perfusion and alters intraglomerular and tubulointerstitial hemodynamics, thereby exacerbating renal damage (Ameer, 2022). In addition, nicotine activates the renin-angiotensin-aldosterone system (RAAS), promoting hypertension, glomerular hyperfiltration, and proteinuria, all of which contribute to progressive renal dysfunction (Ameer, 2022; Oakes et al., 2018). Collectively, these interconnected mechanisms highlight the complex pathophysiological effects of nicotine on the renal microenvironment. However, the precise molecular pathways linking nicotine exposure to podocyte injury and CKD progression remain incompletely understood. Elucidating these mechanisms is essential for identifying novel therapeutic targets and developing effective interventions to prevent or slow smoking-related kidney disease.

Membrane rafts (MRs) are specialized cholesterol- and sphingolipid-rich microdomains within the plasma membrane that function as dynamic signalling platforms, spatially organizing receptors and signalling molecules to regulate diverse cellular processes (Codini et al., 2021). A growing body of evidence indicates that MRs are critical regulators of redox signalling by facilitating the assembly and activation of membrane-associated signalling complexes in response to extracellular stimuli (Patel and Insel, 2009). Upon stimulation, these microdomains undergo dynamic clustering to form membrane raft redox signalling platforms that promote the recruitment and assembly of NADPH oxidase subunits, together with receptors, adaptor proteins, and regulatory molecules. This coordinated assembly triggers NADPH oxidase activation, leading to reactive oxygen species (ROS) generation and the initiation of downstream redox-sensitive signalling cascades that regulate both physiological and pathological cellular responses (Jin et al., 2011; Yi et al., 2009; Li et al., 2007). Consequently, dysregulation of MR redox signalling has emerged as an important mechanism contributing to oxidative stress, inflammation, and tissue injury in a variety of diseases. Our previous work demonstrated that nicotine activates the Nlrp3 inflammasome in podocytes, resulting in inflammasome activation and podocyte injury (Singh et al., 2019). However, the upstream molecular mechanisms responsible for nicotine-induced Nlrp3 inflammasome activation remain poorly defined. Although several pathways have been implicated in Nlrp3 inflammasome activation, including lysosomal destabilization, potassium efflux, mitochondrial dysfunction, and excessive ROS generation is widely regarded as a common upstream signal that links diverse cellular stressors to Nlrp3 inflammasome activation (Koka et al., 2023; Abais et al., 2015; Kelley et al., 2019). Given the established role of membrane raft-associated NADPH oxidase as a major source of ROS, we hypothesized that membrane raft redox signalling serves as a critical upstream mechanism linking nicotine exposure to Nlrp3 inflammasome activation and subsequent podocyte injury. Therefore, the present study tested whether activation of the membrane raft redox signalling pathway mediates nicotine-induced Nlrp3 inflammasome activation and contributes to podocyte dysfunction.

## Materials and methods

### Cell culture

The mouse podocyte cell line employed in our study had been generously provided by Dr. Klotman PE from the Division of Nephrology, Department of Medicine, Mount Sinai School of Medicine, New York, NY, USA, and had been conditionally immortalized (Koka et al., 2023). These cells were cultured on collagen I-coated flasks or plates in RPMI 1640 medium supplemented with recombinant mouse interferon-γ, maintained at a temperature of 33 °C. Following a differentiation period at 37 °C for 10–14 days without interferon–γ, the podocytes were utilized for our experiments. In the experimental design, various treatments involving nicotine alone and in combination with the MR disruptor MCD, the NADPH oxidase inhibitor DPI, caspase-1 inhibitor WEHD or the Asm inhibitor Amitriptyline were administered. The concentrations and incubation durations of these substances in the cell culture dishes were determined based on insights from our previous research and certain preliminary experiments (Singh et al., 2019; Boini et al., 2010; Datta et al., 2024; Datta et al., 2025; Rahman et al., 2025).

### Immuno-fluorescent staining and confocal microscopy

The co-localization of inflammasome molecules in podocytes was investigated by fixing the cultured cells in 4% paraformaldehyde (PFA) for 15 min, followed by rinsing with phosphate-buffered saline (PBS). A blocking step with 1% bovine serum albumin (BSA) was conducted for 30 min. Alexa488-labeled cholera toxin B (CTXB; 1 μg/mL, Thermo Fisher Scientific, USA) was applied for one hour to stain membrane rafts (MRs). The co-localization of MRs with gp91phox or p47phox was examined by incubating podocytes overnight at 4 °C with primary antibodies: anti-gp91phox (1:200, Santa Cruz, USA) or anti-p47phox (1:200, Santa Cruz, USA) for mouse antibodies. For assessing co-localization of inflammasome molecules, specific primary antibodies were used: goat anti-Nlrp3 (1:100, Abcam, Cambridge, MA, USA) with mouse anti-ASC (1:100, Enzo, Plymouth Meeting, PA), or mouse anti-caspase-1 (1:100, Abcam, Cambridge, MA, USA). To investigate the podocyte injury, podocytes were incubated overnight at 4 °C with rabbit anti-podocin (1:200) or rabbit anti-desmin (1:100). On the next day slides were incubated with Alexa-488- or Alexa-555-labeled secondary antibodies for 1 h at room temperature. The slides were then mounted with a DAPI-containing mounting solution and imaged using a confocal laser scanning microscope (Leica). Colocalization analysis, quantified by the Pearson correlation coefficient (PCC), was performed using FIJI ImageJ software for Nlrp3 with ASC or caspase-1 and MR with gp91phox or p47phox. Additionally, the intensity of podocin and desmin expression was analysed using FIJI ImageJ software.

### Electronic spin resonance (ESR) analysis of $O_{2^{.-}}$ production

Superoxide ( $O_{2^{.-}}$ ) production was measured using electron spin resonance (ESR). Proteins extracted from podocytes were resuspended in a modified Kreb’s-Hepes buffer containing 100 mM deferoxamine and 5 mM diethyldithiocarbamate (Sigma-Aldrich). To evaluate NADPH-dependent $O_{2^{.-}}$ production, 1 mM NADPH was added to 20 µg of protein, and the mixture was incubated at 37 °C for 15 min in the presence or absence of 200 U/mL superoxide dismutase (SOD). Following incubation, 1 mM of the $O_{2^{.-}}$ -specific spin trap 1-hydroxy-3-methoxycarbonyl-2,2,5,5-tetramethylpyrrolidine (CMH, Noxygen, Elzach, Germany) was added to the reaction. The samples were loaded into glass capillaries and analyzed for $O_{2^{.-}}$ production kinetics over a 10-min period using a Miniscope MS5000 ESR spectrometer (Magnettech Ltd., Berlin, Germany) (Koka et al., 2023). NADPH-dependent $O_{2^{.-}}$ production was quantified and expressed as fold change relative to control samples.

### Caspase-1 activity and IL-1β production

Following the treatment with nicotine and in combination with the MR disruptor MCD, the NADPH oxidase inhibitor DPI, caspase-1 inhibitor WEHD or the Asm inhibitor Amitriptyline, the podocytes were harvested, and their proteins were extracted through homogenization for the caspase-1 activity assay using a commercially available kit from Biovision. The resulting data were expressed as fold change compared to control cells, enabling us to assess the impact of the treatments on caspase-1 activity (Zhang et al., 2012). Additionally, the cell supernatant was collected to measure IL-1β production. For this purpose, a mouse IL-1β ELISA kit from Bender Medsystems, Burlingame, CA, was employed, following the protocol provided by the manufacturer. This allowed us to determine the levels of IL-1β produced by the podocytes in response to the different treatments.

### Cell permeability assay

Podocytes were cultured onto the upper chambers of 0.4 μm polycarbonate transwell filters in a 24-well filtration microplate (Whatman Inc.). Upon reaching confluence, the culture medium was replaced with fresh serum-free RPMI 1640 media containing nicotine alone and/or in combination with the MR disruptor MCD, caspase-1 inhibitor WEHD, the NADPH oxidase inhibitor DPI, or Asm inhibitor Amitriptyline, and incubate the cells overnight. Subsequently, the media in the upper chambers were replaced with fresh phenol red-free RPMI 1640, and 70 kDa FITC-dextran (2.5 μmol/L) was added to the upper chambers, followed by a 2.5-h incubation period. After incubation, the filtration microplate was removed, and the medium from the lower compartment was collected for analysis. Fluorescence intensity was measured using a spectrofluorometer with excitation at 494 nm and emissions at 521 nm. The obtained relative permeable fluorescence intensity served as a representation of cell permeability in response to the different treatments (Boini et al., 2010).

### Apoptotic index quantification using flow cytometry

Murine podocytes were exposed to nicotine with or without amitriptyline, WEHD, DPI and MCD, overnight. Following treatment, cells were detached using trypsin, collected, and analyzed for early and late apoptotic populations. Supernatants were also collected along with trypsinized cells to assess necrotic populations. For all treatment groups, cells were resuspended in 1X Annexin V binding buffer (Abcam, Cambridge, CA, USA) and stained with Annexin V-FITC and propidium iodide (Abcam, Cambridge, CA, USA). The stained samples were incubated in the dark at room temperature for 5 min before being transferred into sterile flow tubes equipped with 35 µm strainer caps (Southern Labware, Cumming, GA, USA). Apoptotic and necrotic cell populations were then analyzed using a flow cytometer (Accuri C6 Plus cytometer). The percentage of apoptotic cells (early apoptotic + late apoptotic + necrotic populations) were quantified as fold change relative to the control group.

### Statistical analysis

The data is expressed as the arithmetic mean ± standard error of the mean (SEM), with ‘n' representing the number of distinct experiments. Significance testing was carried out using analysis of variance (ANOVA) followed by a *post hoc* test or unpaired Student's t-tests. Results were considered statistically significant if the p-value was less than 0.05.

## Results

### Nicotine induces membrane raft clustering in podocytes

**FIGURE 1:**
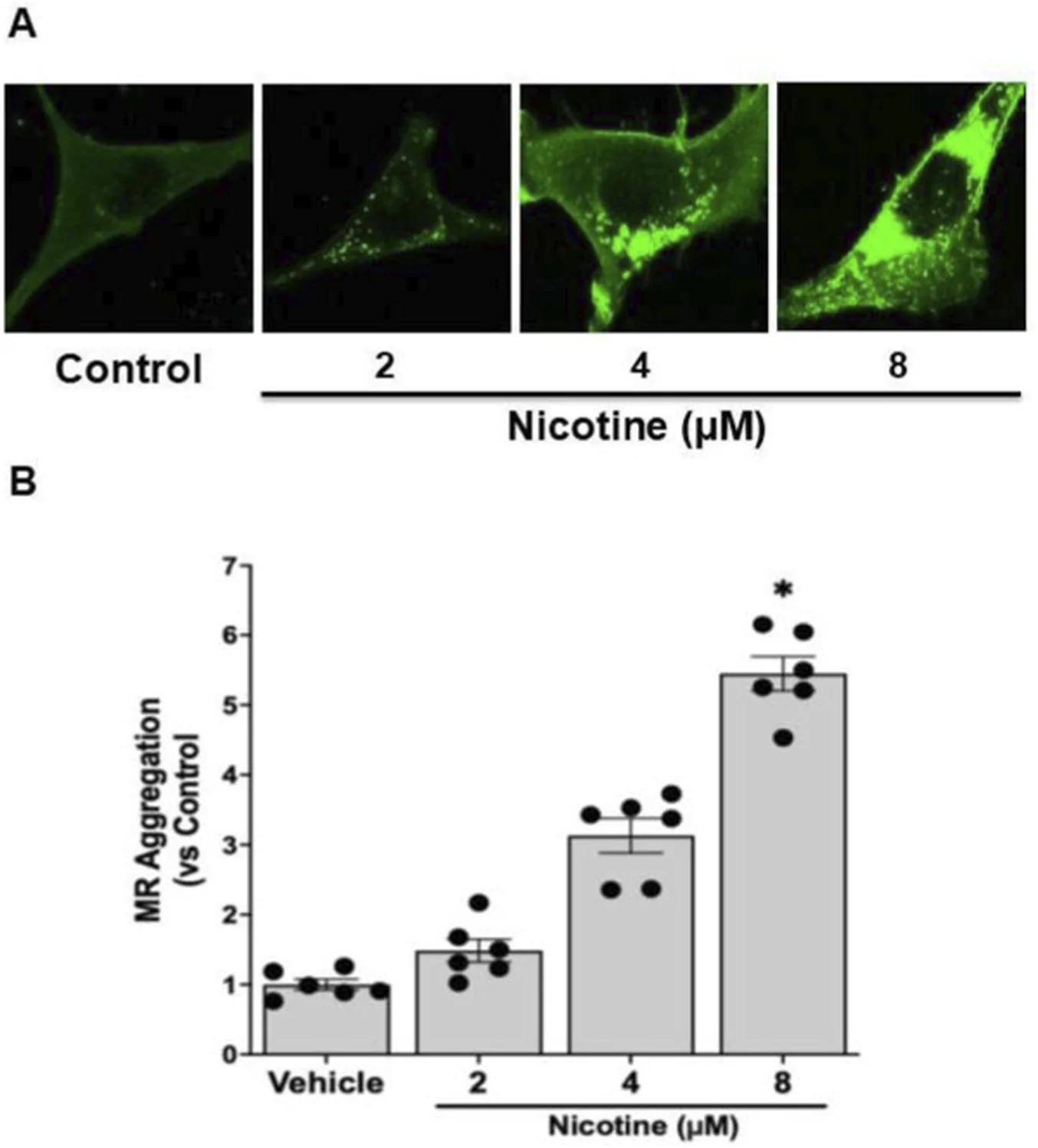
Nicotine causes membrane raft clustering in podocytes. Representative immunofluorescence images **(A)** and summarized quantification data **(B)** show Membrane Raft (MR) Clustering in podocytes with different concentration of Nicotine (2 μM, 4 μM and 8 μM). Images were quantified using ImageJ software. *P < 0.05 vs. control.

As illustrated in Figure 1, to investigate the effects of nicotine on membrane raft (MR) clustering in podocytes, cultured podocytes were treated with increasing concentrations of nicotine (2, 4, and 8 μM) for 16 h. Nicotine treatment induced a dose-dependent increase in MR clustering in podocytes (Figures 1A,B). Based on these findings, 8 μM nicotine was selected as the optimal concentration for subsequent experiments.

### Exposure to nicotine leads to the colocalization of p47phox and gp91phox with membrane raft (MR) clusters

**FIGURE 2:**
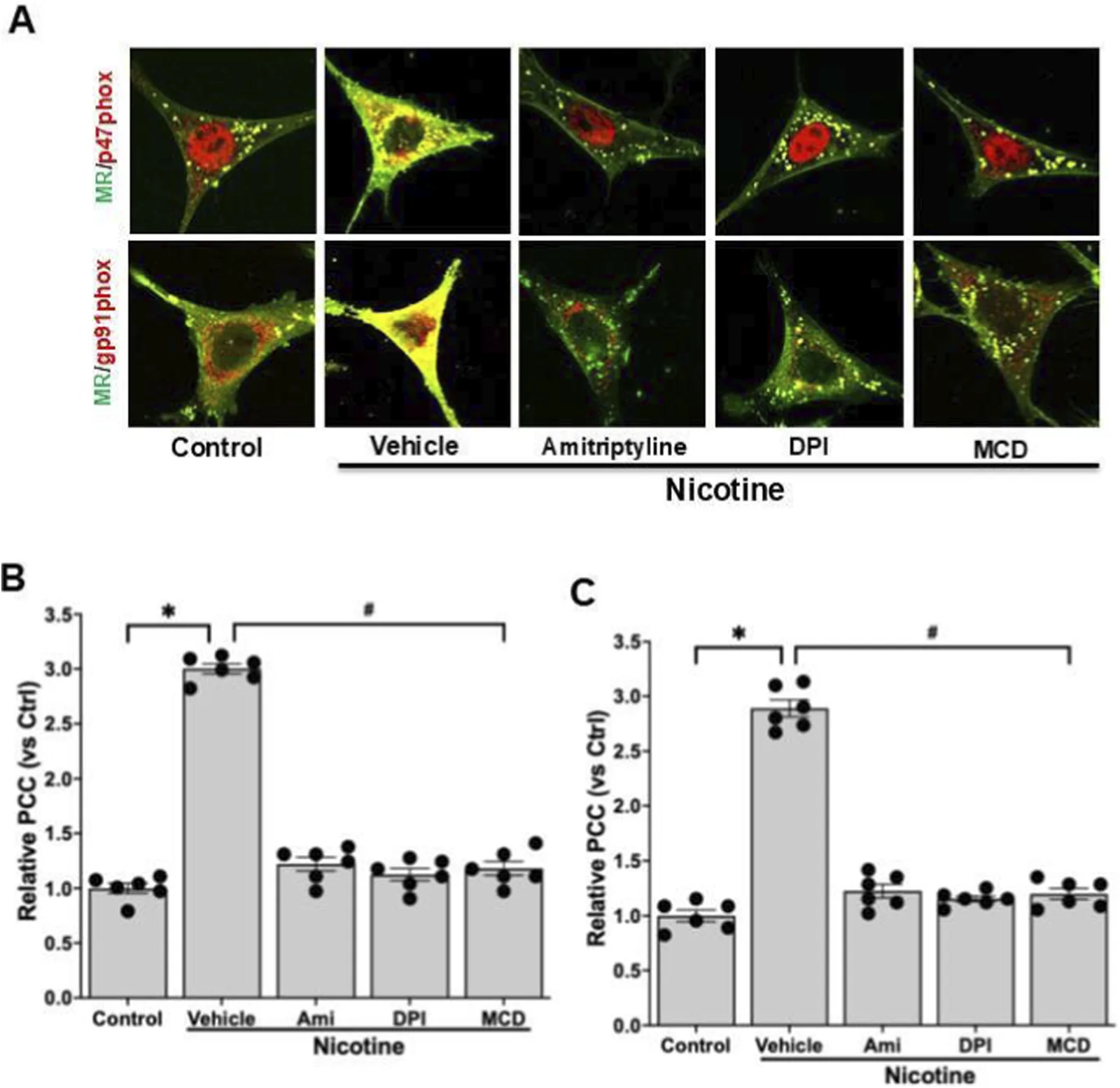
Exposure to nicotine leads to the colocalization of p47phox and gp91phox with membrane raft (MR) clusters in podocytes. Confocal microscopy reveals representative images of membrane raft (MR) clusters (green) and their co-localization with gp91phox (red) or p47phox (red) in podocytes. Merged images highlight co-localization, indicated by yellow spots, between MR clusters and gp91phox or p47phox **(A)**. Quantitative data show the impact of nicotine, the Asm inhibitor amitriptyline, the MR disruptor methyl-β-cyclodextrin (MCD), and the NADPH oxidase inhibitor diphenyleneiodonium (DPI) on MR-p47phox clustering **(B)** and MR-gp91phox clustering **(C)**. Images were analyzed using ImageJ software. Data are presented as fold changes relative to the control group (mean ± SEM). *P < 0.05 vs. control; ^#^P < 0.05 vs. nicotine.

To evaluate the association of gp91phox and p47phox with MRs, we employed antibodies targeting gp91phox or p47phox, along with Alexa Fluor 488-labeled CTXB for co-localization analysis with MRs in podocyte cells. As illustrated in Figure 2A, CTXB displayed a uniform distribution on the podocyte cell membrane in vehicle treated cells. However, upon exposure to nicotine, MRs became prominently visible as intense and deep green fluorescent areas or spots, co-localizing with both p47phox and gp91phox. Subsequently, to assess the significance of MR clusters in facilitating the addition of NADPH oxidase subunits, we examined the impact of the MR disruptor MCD on nicotine-induced effects. Our results indicated a significant reduction in the co-localization of p47phox and gp91phox with MRs in the presence of MCD. Furthermore, we investigated the influence of DPI, a NADPH oxidase inhibitor, and amitriptyline, an asm inhibitor, on the formation of gp91phox and p47phox aggregates within MR clusters. The findings demonstrated a marked decrease in the co-localization of gp91phox or p47phox with MRs in the presence of DPI and amitriptyline, suggesting a feedback regulatory mechanism governing the clustering of membrane rafts (MRs) (Figures 2B,C).

### Nicotine induces $O_{2^{.-}}$ production via NADPH oxidase activation

**FIGURE 3:**
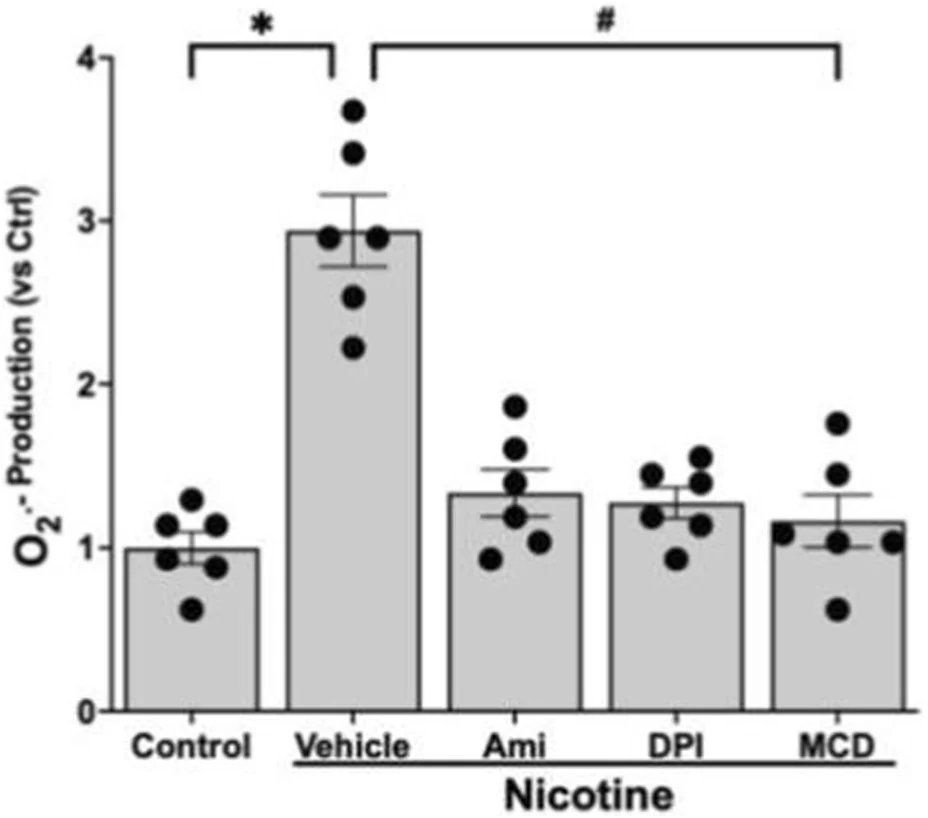
Nicotine induces $O_{2^{.-}}$ production via NADPH oxidase activation and its inhibition attenuates the effect. The summarized data highlight the effects of nicotine on $O_{2^{.-}}$ production in podocytes and the impact of treatments with amitriptyline, MCD, and DPI. Data are expressed as fold changes relative to the control group (mean ± SEM). *P < 0.05 vs. control; ^#^P < 0.05 vs. nicotine.

To elucidate the contribution of NADPH oxidase to nicotine-induced superoxide (O_2_•^-^) generation, we assessed reactive oxygen species production using electron spin resonance (ESR) spectroscopy. Nicotine exposure significantly increased superoxide levels in podocytes compared with vehicle-treated controls (Figure 3). Notably, pretreatment with the membrane raft (MR) disruptor MCD, the NADPH oxidase inhibitor DPI, or the acid sphingomyelinase (Asm) inhibitor amitriptyline significantly attenuated the nicotine-induced superoxide production, restoring levels to those observed in control cells. These findings demonstrate that nicotine-induced oxidative stress in podocytes is mediated through an MR-dependent NADPH oxidase signalling pathway, highlighting membrane raft-associated redox activation as a key mechanism driving nicotine-induced podocyte dysfunction.

### Blocking NADPH oxidase reduced the Nlrp3 inflammasomes formation caused by nicotine

**FIGURE 4:**
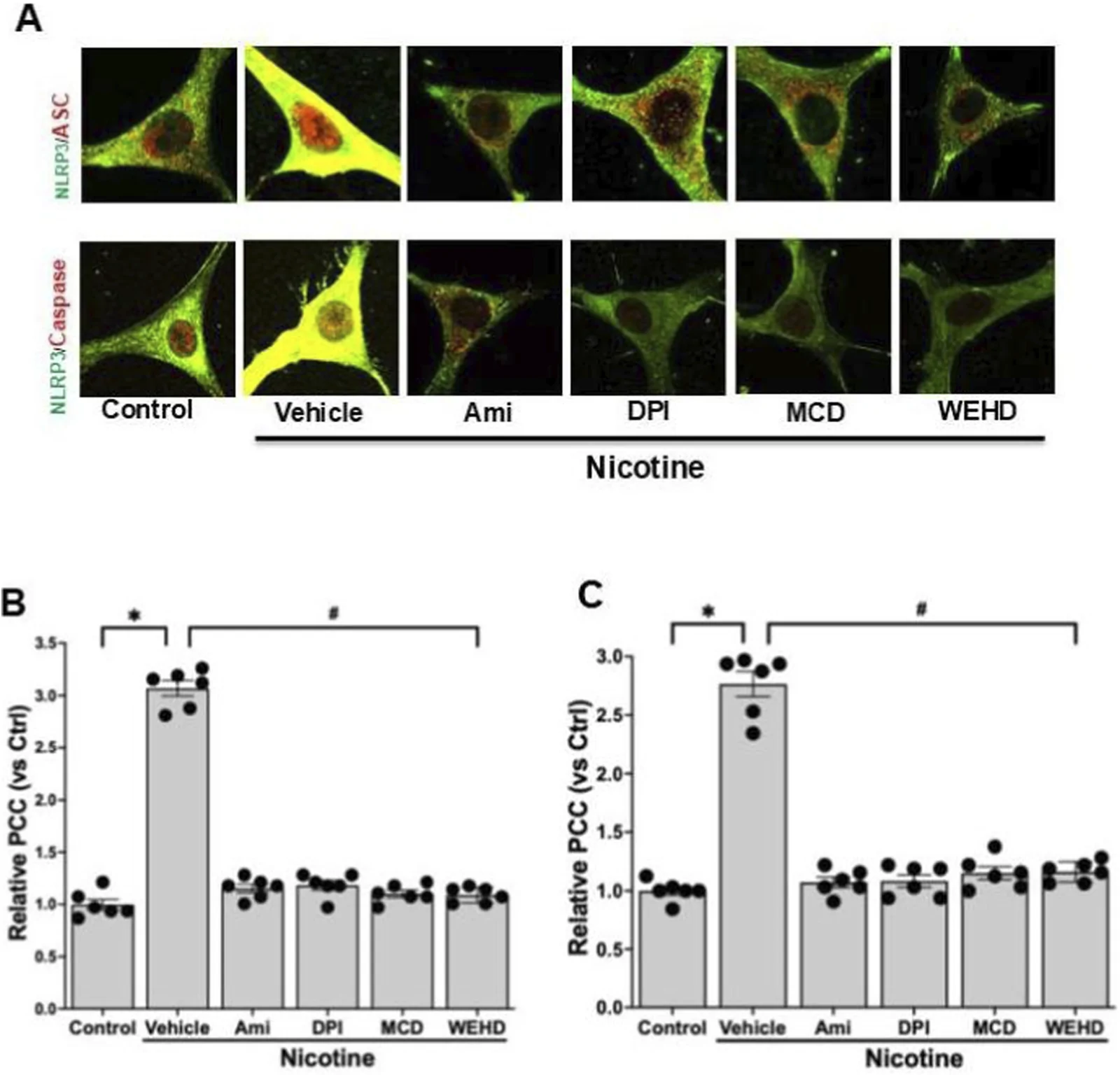
Blocking NADPH oxidase reduced the formation and activation of inflammasomes caused by nicotine. Confocal images showing the colocalization of Nlrp3 (green) with ASC (red) and Nlrp3 (green) with caspase-1 (red) in podocytes **(A)**. Quantitative analysis of the Pearson correlation coefficient (PCC) for the colocalization of NLRP3 with ASC **(B)** and NLRP3 with caspase-1 **(C)** is presented. Images were analyzed using ImageJ software. Data are expressed as fold changes relative to the control group (mean ± SEM). *P < 0.05 vs. control; ^#^P < 0.05 vs. nicotine.

Next, we examined whether nicotine-induced NADPH oxidase activation promotes podocyte injury through the induction of Nlrp3 inflammasome assembly and activation. To determine whether nicotine stimulates inflammasome formation in podocytes, we examined the co-localization of key Nlrp3 inflammasome components using confocal microscopy. Nicotine treatment significantly increased the co-localization of Nlrp3 with ASC and Nlrp3 with caspase-1, indicating robust inflammasome assembly and activation. Pretreatment with the membrane raft (MR) disruptor MCD, the NADPH oxidase inhibitor diphenyleneiodonium (DPI), or the acid sphingomyelinase (Asm) inhibitor amitriptyline markedly reduced nicotine-induced Nlrp3 with Asc and Nlrp3 with caspase-1 co-localization (Figure 4), thereby preventing inflammasome complex formation. These findings demonstrate that nicotine activates the Nlrp3 inflammasome through an MR-dependent NADPH oxidase signaling pathway. Collectively, our results identify membrane raft-associated redox signaling as an upstream mechanism linking nicotine exposure to inflammasome activation and podocyte injury and suggest that targeting NADPH oxidase-mediated signaling may provide a potential therapeutic strategy to limit nicotine-induced renal damage.

### Inhibition of NADPH oxidase reduced nicotine-induced Nlrp3 inflammasome activation

**FIGURE 5:**
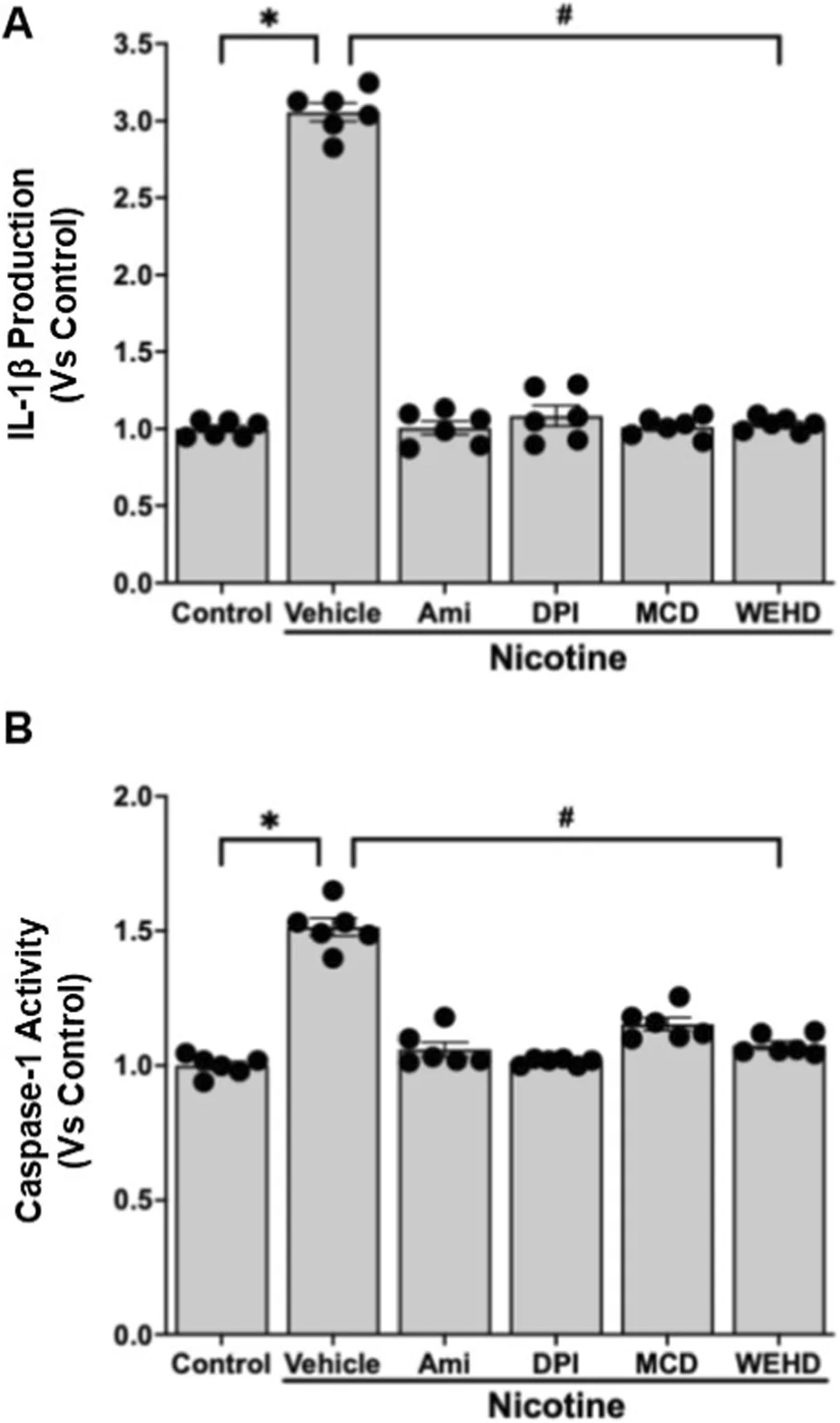
Suppressing NADPH oxidase led to a decrease in the activation of the Nlrp3 inflammasome triggered by Nicotine. IL-1β production **(A)** and Caspase-1 activity **(B)** in podocytes were assessed under conditions of nicotine stimulation, with or without the addition of amitriptyline, DPI, or MCD. Data are expressed as fold changes relative to the control group (mean ± SEM). *P < 0.05 vs. control; ^#^P < 0.05 vs. nicotine.

Nicotine treatment significantly increased IL-1β production (Figure 5A) and caspase-1 activity (Figure 5B) in podocytes compared with vehicle-treated controls. Consistent with Nlrp3 inflammasome activation, nicotine promoted the conversion of pro-caspase-1 to its active form, leading to the maturation and release of the pro-inflammatory cytokine IL-1β. Notably, pretreatment with the NADPH oxidase inhibitor DPI or membrane raft (MR) disruptor MCD or the acid sphingomyelinase (Asm) inhibitor amitriptyline markedly attenuated nicotine-induced caspase-1 activation and IL-1β production. These findings indicate that NADPH oxidase-derived oxidative stress is an upstream regulator of nicotine-induced Nlrp3 inflammasome activation in podocytes.

### Blocking Nlrp3 inflammasome activation or NADPH oxidase signaling reduces nicotine-induced podocyte injury

**FIGURE 6:**
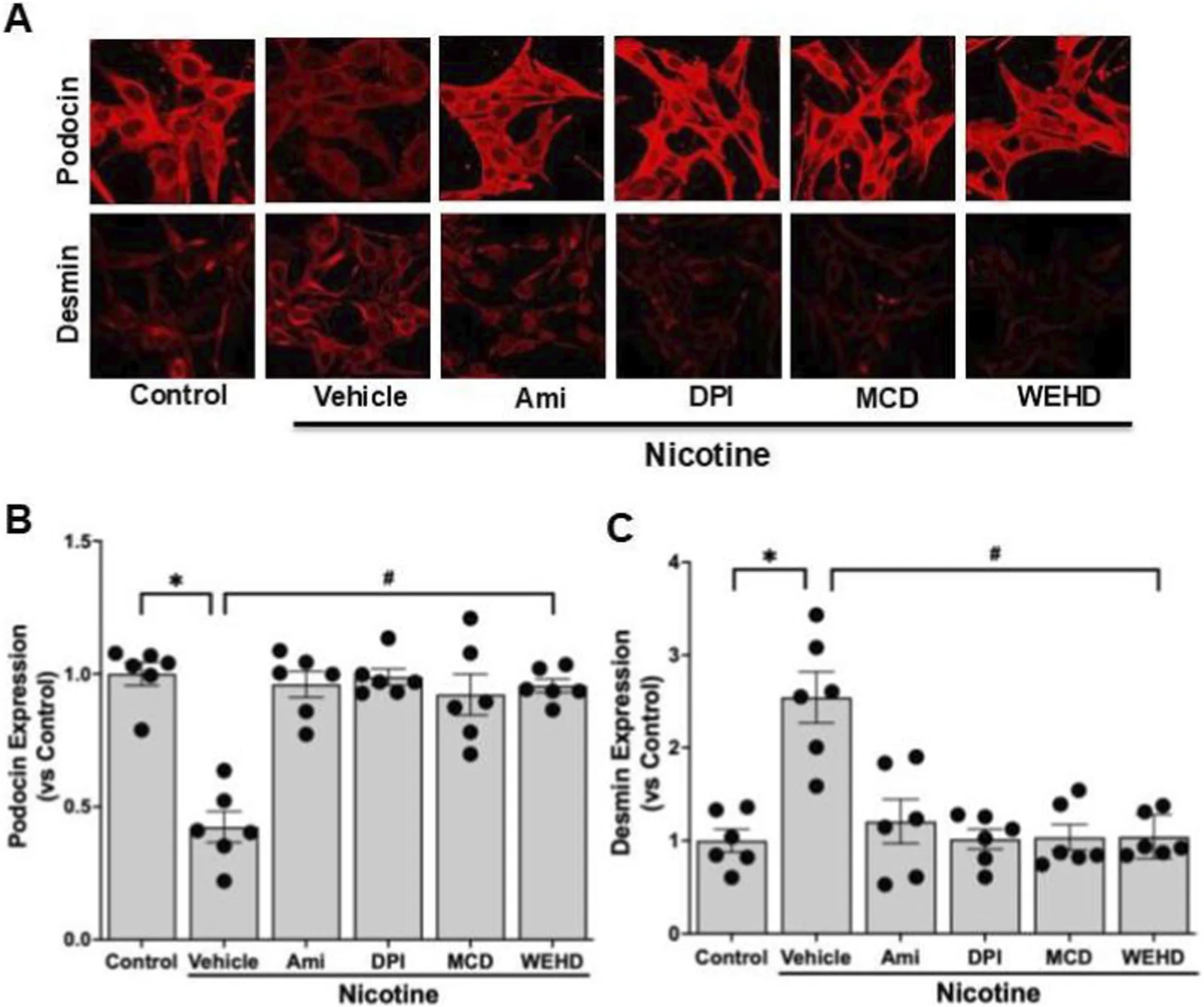
Blocking inflammasomes or NADPH oxidase lessens the damage to podocytes caused by nicotine. Confocal image analysis revealed that nicotine treatment significantly decreased the expression of podocin and increased desmin expression in podocytes **(A)**. Treatment with amitriptyline, WEHD, DPI, or MCD significantly reversed these effects **(B,C)**. Quantification of the images was performed using ImageJ software. Data are expressed as fold changes relative to the control group (mean ± SEM). *P < 0.05 vs. control; ^#^P < 0.05 vs. nicotine.

To evaluate nicotine-induced podocyte injury, we assessed the expression of key slit diaphragm proteins, including podocin and desmin (Figure 6A). Podocin, a podocyte-specific structural protein, is typically reduced during podocyte injury, whereas increased desmin expression serves as a marker of podocyte damage. Using confocal microscopy, we examined the effects of nicotine exposure and pharmacological inhibition of the associated signaling pathways on podocyte integrity. Nicotine treatment resulted in a marked reduction in podocin expression and a significant increase in desmin staining, indicating disruption of podocyte structure and injury. Importantly, pretreatment with the membrane raft (MR) disruptor MCD, the NADPH oxidase inhibitor DPI, or the acid sphingomyelinase (Asm) inhibitor amitriptyline, caspase-1 inhibitor WEHD significantly preserved podocin expression and reduced desmin accumulation, restoring levels toward those observed in control cells (Figures 6B,C). These findings demonstrate that inhibition of MR-associated NADPH oxidase/Asm signaling protects podocytes from nicotine-induced structural damage. Collectively, our results suggest that NADPH oxidase-dependent redox signaling acts as an upstream mechanism linking nicotine exposure to Nlrp3 inflammasome activation and podocyte dysfunction.

### NADPH oxidase inhibitors prevent nicotine-induced podocyte barrier disruption and increased monolayer permeability

**FIGURE 7:**
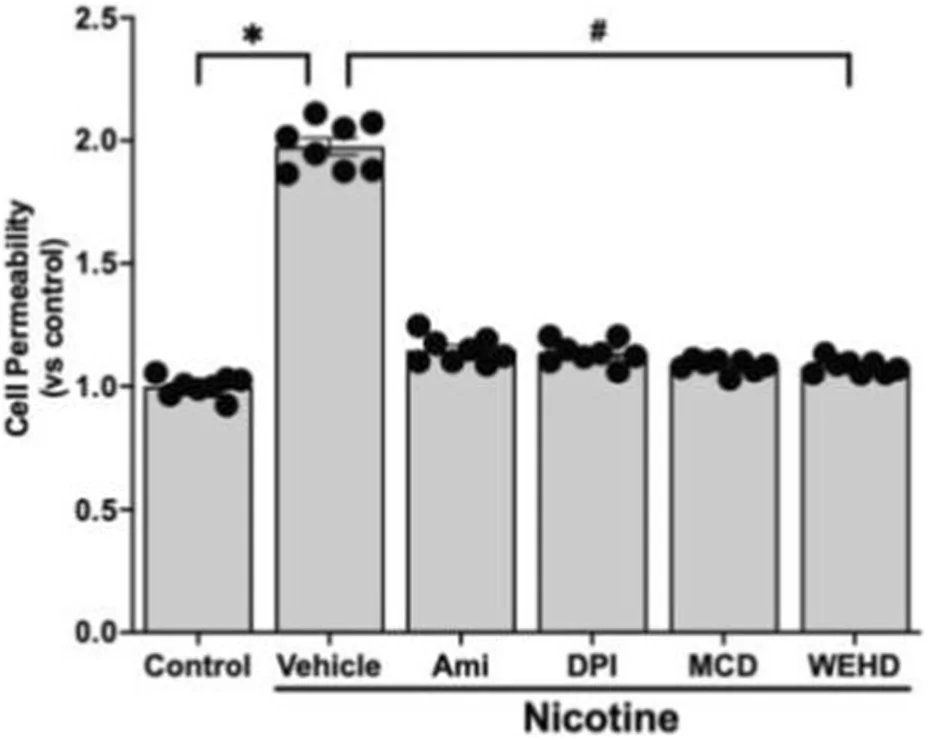
Protective effect of NADPH oxidase inhibitors against nicotine-induced podocyte dysfunction and increased monolayer permeability. Cell permeability in podocytes treated with nicotine, with or without amitriptyline, WEHD, MCD, or DPI, was evaluated. Values represent arithmetic means ± SEM. Data are expressed as fold changes relative to the control group (mean ± SEM). *P < 0.05 vs. control; ^#^P < 0.05 vs. nicotine.

Furthermore, we investigated the functional consequences of Nlrp3 inflammasome activation on nicotine-induced alterations in podocyte barrier function by assessing monolayer permeability using FITC-dextran flux analysis. Nicotine exposure significantly increased podocyte monolayer permeability compared with untreated controls (Figure 7), indicating impaired barrier integrity. Notably, pretreatment with the membrane raft (MR) disruptor MCD, the NADPH oxidase inhibitor DPI, or the acid sphingomyelinase (Asm) inhibitor amitriptyline markedly attenuated nicotine-induced increases in permeability (Figure 7). These findings demonstrate that nicotine-induced disruption of podocyte barrier function is mediated through MR-associated NADPH oxidase/Asm signalling pathway.

### Inhibition of NADPH oxidase or asm recovers nicotine-induced podocyte apoptosis

**FIGURE 8:**
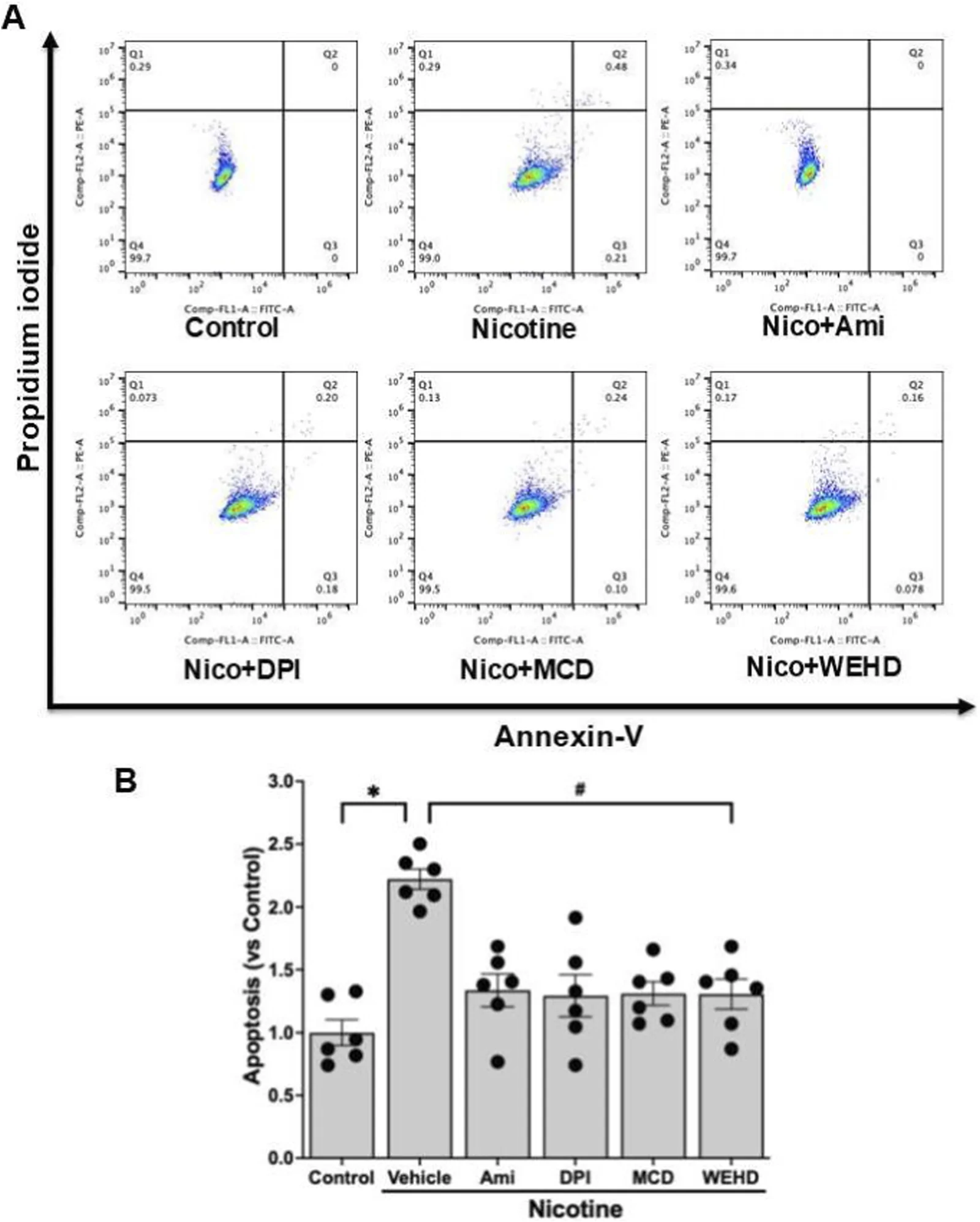
Inhibition of Asm, MR, inflammasome and NADPH oxidase protects against nicotine-induced apoptosis in podocytes. Flow cytometry analysis **(A)** and corresponding quantification **(B)** were performed to investigate the role of different inhibitors in nicotine-mediated podocyte apoptosis. Data were analyzed using FlowJo v10.10.0 software, with apoptotic cells (%) calculated as the sum of early apoptotic, late apoptotic, and necrotic populations. Results are presented as fold change relative to the control group. *p < 0.05 vs. control group, *p* < 0.05 vs. nicotine-treated group; ^#^ significant difference from the nicotine-treated group. Q1: necrotic cells, Q2: late apoptotic cells, Q3: early apoptotic cells, Q4: live cells, Ctrl: control podocytes, Nico: nicotine (8 µM)-treated podocytes.

We investigated the effects of nicotine exposure on podocyte apoptosis in the presence or absence of the acid sphingomyelinase (Asm) inhibitor amitriptyline or MCD or DPI. Flow cytometric analysis demonstrated that nicotine treatment significantly increased the populations of late apoptotic (Q2) cells compared with vehicle-treated podocytes (Figure 8A), indicating enhanced cell death following nicotine exposure. Importantly, pretreatment with amitriptyline, the caspase-1 inhibitor WEHD, the NADPH oxidase inhibitor DPI, or the membrane raft (MR) disruptor MCD or acid sphingomyelinase inhibitor amitriptyline markedly reduced nicotine-induced apoptotic and necrotic cell populations (Figure 8B). These findings demonstrate that nicotine-induced podocyte apoptosis is mediated through an MR-dependent NADPH oxidase/Asm/Nlrp3 inflammasome signalling pathway. Pharmacological inhibition of Asm or upstream redox signalling components effectively protects podocytes from nicotine-induced cell death, highlighting this pathway as a potential therapeutic target for preventing nicotine-associated podocyte injury.

## Discussion

The present study aimed to elucidate the role of membrane raft associated redox signalling in nicotine-induced Nlrp3 inflammasome activation and subsequent podocyte injury. Using cultured podocytes as an experimental model, we identified a previously unrecognized and critical function of MR redox signalling in regulating the assembly and activation of the Nlrp3 inflammasome upon nicotine exposure. Our findings reveal a mechanistic link between nicotine stimulation, membrane raft redox signalling, and inflammasome activation, providing new insights into the molecular pathways driving podocyte dysfunction. These results highlight MR redox signalling as a key mechanism in nicotine-mediated renal injury and suggest that targeting membrane raft-associated oxidative pathways or Nlrp3 inflammasome activation may represent a potential therapeutic strategy for preventing nicotine-associated kidney damage.

Nicotine, the primary psychoactive constituent of tobacco, exerts extensive detrimental effects on both renal and cardiovascular systems, establishing a strong association between tobacco exposure and systemic disease. In the kidney, nicotine induces renal hemodynamic alterations by promoting renal vasoconstriction, thereby reducing renal perfusion and impairing glomerular filtration (Singh et al., 2013). In addition, nicotine-mediated activation of inflammatory pathways and increased oxidative stress contribute significantly to renal injury and the progression of chronic kidney disease (CKD) (Jain and Jaimes, 2013). Similarly, the cardiovascular consequences of nicotine exposure are well established, including increased heart rate, blood pressure, and systemic vascular resistance, which collectively enhance the risk of hypertension, atherosclerosis, and cardiovascular disease (Espinoza-Derout et al., 2022). Nicotine activates the sympathetic nervous system, resulting in catecholamine release and subsequent increases in cardiac activity and vascular tone (Benowitz and Burbank, 2016). Moreover, nicotine stimulates the production of vasoactive factors such as endothelin-1, promoting vasoconstriction and vascular resistance (Whitehead et al., 2021). Chronic nicotine exposure further exacerbates endothelial dysfunction, inflammation, oxidative stress, and atherosclerotic progression (Li et al., 2018). Although the adverse effects of nicotine on renal function are well recognized, the precise molecular mechanisms underlying nicotine-induced podocyte injury remain poorly defined. Previous studies from our laboratory demonstrated that nicotine directly impairs podocyte function (Rahman et al., 2025). Specifically, nicotine exposure reduced podocin expression, a key marker of podocyte integrity, while increasing desmin expression, resulting in enhanced podocyte permeability and structural dysfunction. Furthermore, our recent findings revealed that nicotine markedly increases superoxide production in podocytes, inducing oxidative stress and disrupting cellular permeability, thereby contributing to renal injury (Singh et al., 2019; Datta et al., 2024; Datta et al., 2025). However, the upstream signalling mechanisms responsible for initiating nicotine-induced oxidative stress, inflammasome activation, and podocyte damage remain unclear.

Membrane rafts (MRs), cholesterol- and sphingolipid-enriched microdomains within the plasma membrane, function as specialized signalling platforms that regulate redox-sensitive inflammatory pathways. Therefore, the present study investigated whether nicotine activates MR-associated redox signalling and whether this pathway contributes to Nlrp3 inflammasome activation and podocyte injury. Cultured podocytes were exposed to increasing concentrations of nicotine (2, 4, and 8 μM), resulting in a dose-dependent increase in MR aggregation compared with untreated controls. These findings demonstrate that nicotine promotes MR reorganization and activation, suggesting a novel role for MR-associated redox signalling as an upstream mechanism linking nicotine exposure to oxidative stress, inflammasome activation, and podocyte dysfunction.

Our study further demonstrated that nicotine treatment significantly enhanced the co-localization of membrane raft (MRs) with the NADPH oxidase subunits gp91^phox^ and p47^phox^ in podocytes compared with control cells. Nicotine-induced MR clustering and subsequent NADPH oxidase activation were markedly attenuated by MR disruptor MCD or inhibition of NADPH oxidase activity with DPI, or inhibition of acid sphingomyelinase (Asm) activity with amitriptyline. These findings indicate that nicotine promotes the recruitment and aggregation of NADPH oxidase components within MR domains, facilitating the assembly of an active oxidase complex and establishing MR-dependent redox signalling as an initiating event in nicotine-induced podocyte injury (Figure 2).

To further characterize the contribution of MR-associated NADPH oxidase clustering to oxidative stress, electron spin resonance (ESR) was employed to quantify NADPH oxidase-derived superoxide production. Nicotine exposure resulted in a significant increase in superoxide generation, which was effectively prevented by MCD, DPI, or amitriptyline pretreatment. These findings demonstrate that nicotine induces oxidative stress through MR-dependent assembly and activation of NADPH oxidase complexes. Disruption of MR organization or inhibition of NADPH oxidase and Asm signalling effectively suppressed ROS production, highlighting the essential role of this pathway in nicotine-mediated podocyte dysfunction.

We next investigated whether nicotine-induced MR-dependent redox signalling contributes to Nlrp3 inflammasome activation. The Nlrp3 inflammasome is a central regulator of innate immune responses and has been implicated in numerous inflammatory and degenerative disorders (Holbrook et al., 2021; Anderson et al., 2023; Li et al., 2021). In podocytes, nicotine stimulation significantly increased IL-1β production and promoted the assembly of Nlrp3 inflammasomes, evidenced by enhanced co-localization of Nlrp3 with ASC and Nlrp3 with caspase-1. Importantly, nicotine-induced inflammasome assembly and activation were substantially inhibited by pretreatment with MCD, DPI, or amitriptyline (Figures 4, 5). To further validate inflammasome activation, caspase-1 activity was assessed as a functional indicator of Nlrp3 activation. Nicotine treatment markedly increased caspase-1 activity, whereas disruption of MR integrity, inhibition of NADPH oxidase, or blockade of Asm signalling significantly reduced caspase-1 activation. These findings provide evidence that MR-associated NADPH oxidase activation functions as an upstream regulator of nicotine-induced Nlrp3 inflammasome activation in podocytes.

Furthermore, we determined whether inhibition of the MR/NADPH oxidase/Nlrp3 inflammasome pathway could protect podocytes from nicotine-induced podocyte injury. Nicotine treatment significantly decreased podocin expression, a key marker of podocyte integrity, while increasing desmin expression, indicative of podocyte injury (Figure 6). Remarkably, inhibition of MR disruption, NADPH oxidase activation, or Asm signalling preserved podocyte morphology and maintained expression profiles associated with podocyte functional integrity. These findings suggest that targeting MR-dependent inflammasome activation not only suppresses inflammatory signalling but also protects podocyte structure and function. Together, these results identify a previously unrecognized MR-associated redox signalling axis in which nicotine promotes NADPH oxidase activation, oxidative stress, Asm signalling, and Nlrp3 inflammasome activation, ultimately leading to podocyte injury. This pathway provides new mechanistic insight into nicotine-induced renal damage and highlights MR-associated redox signalling as a potential therapeutic target for preventing nicotine-associated kidney disease.

In addition, we investigated the functional relevance of nicotine-induced MR-associated redox signalling in Nlrp3 inflammasome activation by evaluating its impact on podocyte monolayer permeability. Increased glomerular permeability is a hallmark of podocyte dysfunction and represents a critical contributor to glomerular injury (Koka et al., 2023). Our findings demonstrated that nicotine exposure significantly increased podocyte permeability, whereas this effect was markedly attenuated by pretreatment with the membrane raft (MR) disruptor MCD, the NADPH oxidase inhibitor DPI, or the Asm inhibitor amitriptyline. These results suggest that nicotine-induced disruption of podocyte barrier function is mediated, at least in part, through activation of the MR–NADPH oxidase–Nlrp3 inflammasome signalling axis (Figure 7). Podocyte apoptosis and loss of barrier integrity are key events contributing to glomerular injury and CKD progression. Our findings demonstrate that nicotine significantly increased podocyte apoptosis and necrotic cell death, while inhibition of the MR/NADPH oxidase/Asm/Nlrp3 pathway markedly preserved podocyte viability (Figure 8). Collectively, these findings further establish the functional importance of MR-dependent redox signalling in linking nicotine exposure to podocyte dysfunction and glomerular injury. Despite these encouraging findings, the present study has several limitations. First, all experiments were conducted using cultured podocytes therefore, our conclusions are limited to an *in vitro* model. Further studies using appropriate animal models are warranted to determine whether the membrane raft-mediated redox signalling mechanisms identified here similarly contribute to nicotine-induced Nlrp3 inflammasome activation and podocyte injury *in vivo*. Second, although our pharmacological data support the involvement of membrane raft-associated NADPH oxidase signalling inhibitors such as DPI and MCD have recognized limitations, including off-target effects and non-specific disruption of membrane-associated signalling pathways. Therefore, future studies employing more selective NOX inhibitors, membrane raft-targeting strategies, or genetic approaches, such as NOX2 (gp91^phox^) or p47^phox^ knockdown or knockout models, will be important to define the specific contribution of NADPH oxidase isoforms and membrane raft signalling to nicotine-induced Nlrp3 inflammasome activation. Third, although nicotine-induced membrane raft recruitment of gp91^phox^ and p47^phox^, together with increased superoxide production, strongly suggests NADPH oxidase activation, direct evidence of enzyme activation was not obtained. Future studies incorporating NADPH oxidase activity assays, analyses of enzyme complex assembly, and genetic manipulation of specific NOX isoforms will be necessary to definitively establish NADPH oxidase activation as the upstream mediator of nicotine-induced membrane raft redox signalling and subsequent Nlrp3 inflammasome activation in podocytes.

In conclusion, our study identifies a novel mechanism by which nicotine induces podocyte injury through activation of membrane raft (MR)-associated redox signalling. Nicotine promotes MR clustering within podocyte membranes, facilitating the recruitment and assembly of NADPH oxidase subunits into functional redox signalling platforms. This process drives oxidative stress, activates the Nlrp3 inflammasome, and ultimately leads to podocyte dysfunction and injury. These findings provide new mechanistic insight into the molecular basis of nicotine-induced renal damage and establish MR-dependent redox signalling as a critical link between nicotine exposure, oxidative inflammation, and podocyte impairment. Targeting MR-associated signalling pathways, NADPH oxidase activation, or downstream inflammasome responses may represent potential therapeutic strategies to mitigate kidney injury associated with nicotine exposure.

## Data Availability

The raw data supporting the conclusions of this article will be made available by the authors, without undue reservation.
